# Proxy methods for detection of inhalation exposure in simulated office environments

**DOI:** 10.1038/s41370-022-00495-w

**Published:** 2022-11-08

**Authors:** Seoyeon Yun, Sailin Zhong, Hamed S. Alavi, Alexandre Alahi, Dusan Licina

**Affiliations:** 1 Human-Oriented Built Environment Lab, School of Architecture, Civil and Environmental Engineering, École Polytechnique Fedérale de Lausanne, Lausanne, Switzerland; 2 Human-IST Institute, Department of Informatics, University of Fribourg, Fribourg, Switzerland; 3 Digital Interactions Lab, Institute of Informatics, University of Amsterdam, Amsterdam, The Netherlands; 4 Visual Intelligence for Transportation, School of Architecture, Civil and Environmental Engineering, École Polytechnique Fedérale de Lausanne, Lausanne, Switzerland

**Keywords:** Personal exposure estimation, Environmental monitoring, Workplace exposures, Carbon dioxide, Particulate matter

## Abstract

**Background:**

Modern health concerns related to air pollutant exposure in buildings have been exacerbated owing to several factors. Methods for assessing inhalation exposures indoors have been restricted to stationary air pollution measurements, typically assuming steady-state conditions.

**Objective:**

We aimed to examine the feasibility of several proxy methods for estimating inhalation exposure to CO_2_, PM_2.5_, and PM_10_ in simulated office environments.

**Methods:**

In a controlled climate chamber mimicking four different office setups, human participants performed a set of scripted *sitting* and *standing* office activities. Three proxy sensing techniques were examined: stationary indoor air quality (IAQ) monitoring, individual monitoring of physiological status by wearable wristband, human presence detection by Passive Infrared (PIR) sensors. A ground-truth of occupancy was obtained from video recordings of network cameras. The results were compared with the concurrent IAQ measurements in the breathing zone of a reference participant by means of multiple linear regression (MLR) analysis with a combination of different input parameters.

**Results:**

Segregating data onto *sitting* and *standing* activities could lead to improved accuracy of exposure estimation model for CO_2_ and PM by 9–60% during *sitting* activities, relative to *combined* activities. Stationary PM_2.5_ and PM_10_ monitors positioned at the ceiling-mounted ventilation exhaust in vicinity of the seated reference participant accurately estimated inhalation exposure (adjusted *R*² = 0.91 and *R*² = 0.87). Measurement at the front edge of the desk near abdomen showed a moderate accuracy (adjusted *R*² = 0.58) in estimating exposure to CO_2_. Combining different sensing techniques improved the CO_2_ exposure detection by twofold, whereas the improvement for PM exposure detection was small (~10%).

**Significance:**

This study contributes to broadening the knowledge of proxy methods for personal exposure estimation under dynamic occupancy profiles. The study recommendations on optimal monitor combination and placement could help stakeholders better understand spatial air pollutant gradients indoors which can ultimately improve control of IAQ.

## Introduction

Indoor air quality (IAQ) is shaped by myriad factors such as ventilation strategy, space type, and outdoor and indoor climatic conditions and air pollution sources [1]. In particular, human presence and activities have considerable impact on spatio-temporal variation of indoor air pollutants such as particulate matter (PM), carbon dioxide (CO_2_), and total volatile organic compounds (TVOCs) [2, 3]. Exposure to elevated levels of metabolically–generated CO_2_ could have implications for several health symptoms (e.g., sneezing, irritated eyes, dry or itchy skin), impaired cognitive functioning and decision making [4, 5]. Similarly, elevated exposure to PM_2.5_ and PM_10_ can increase cumulative incidence of respiratory symptoms such as, throat irritation, coughing, asthma [6].

In office buildings, where occupants are often major contributors to air pollution burden [7], occupancy information could be utilized to characterize IAQ. Occupant activities (e.g., walking, cleaning, and sitting on furniture) cause resuspension of particles, particularly in the coarse (>2.5 μm) size range [8, 9]. Humans also generate bioeffluents including water vapor, CO_2_, and VOCs [10–12]. Thus, occupancy-associated emissions are potent determinants of IAQ and as such, they play a fundamental role in exposure estimation and ventilation control [13, 14].

Inhalation exposure assessment studies performed in controlled chambers typically included steady-state conditions and a fixed occupancy [15–17]. Although steady-state studies can be useful, highly controlled environments give little resemblance with the actual exposures that are encountered in real buildings. Furthermore, in spaces where indoor air is imperfectly mixed, spatial heterogeneity of air pollution represents a challenge for assessing inhalation exposures [18].

The common practice of positioning an IAQ monitor indoors is based on standard recommendations that consider the ergonomics of the thermal environment (e.g., EN ISO 7726:2001, EPA Air Sensor Guidebook) [19, 20]. Researchers typically position CO_2_ monitors in the middle of an occupied zone with heights between 1.0–1.2 m, which falls into a breathing zone height [21]. Other frequently selected locations for examining indoor air pollution include supply/exhaust ventilation grills, walls and office desks [22, 23]; although substantial differences between concentrations recorded from stationary monitors and those recorded in the breathing zone of occupants have been reported [24, 25].

To improve the accuracy of personal air pollution exposure assessment, several studies have combined other methods with IAQ monitoring [26–28]. These studies collected occupancy information through self-reported diaries, staff monitoring or Passive Infrared (PIR) sensors, and correlated them with personal CO_2_ [26, 27] or PM [26, 28] exposures. However, previous studies have not investigated occupancy characteristics (e.g., occupant activities) in order to better characterize inhalation exposure to indoor air pollutants.

As previously noted, methods for detecting personal exposures to CO_2_ and PM under dynamic indoor environments are largely unexplored. To bridge this knowledge gap, our study examined a combination of physical parameters (environmental, contextual, and physiological) which best represents inhalation exposures to CO_2_, PM_2.5_, and PM_10_ in a simulated office environment with dynamic occupancy profiles. Specifically, we performed continuous air quality measurements in the breathing zone of a reference participant with the concurrent IAQ measurement by stationary monitors, occupancy presence by PIR sensors, physiological characteristics of the participant by wearable wristband. A ground-truth occupancy was collected from the network cameras installed in the chamber. A multiple linear regression (MLR) analysis was applied to identify the best proxy methods to detect inhalation exposure to CO_2_, PM_2.5_, and PM_10_. Finally, we proposed the best proxy method(s) for characterizing inhalation exposure for investigated scenarios, which includes information about what parameters to monitor and where.

## Methods

### Chamber description and space layouts

**Fig. 1:**
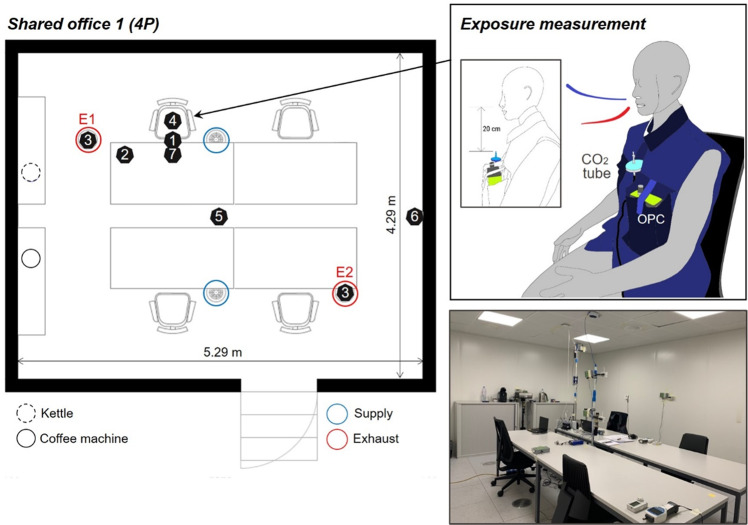
Example of monitor placement in the Shared office 1 (4 participants) and exposure measurement (CO_2_, PM) in the breathing zone of the reference participant. Each monitor location is marked with an ID number which is described in Table 1. Notes: E1 = Exhaust 1, E2 = Exhaust 2.

The experiments were conducted in a controlled climate chamber (floor area: 24.8 m^2^, volume: 60 m^3^), where air temperature and relative humidity were controlled within narrow ranges, 24.9 ± 0.4°C and 54.3 ± 4%, respectively. To simulate typical mechanically-conditioned office spaces, we selected the mixing ventilation strategy, which is the most common air distribution method applied in commercial office buildings [29]. Here, the conditioned air was supplied and exhausted through the two swirl-type diffusers at the ceiling of the chamber (Fig. 1). The air change rate was constant (2.4–2.6 h^−1^), which was confirmed by the CO_2_ tracer gas decay method [30]. The corresponding air change rate matched the recommendation value (ventilation rate of 144–156 m^3^/h for four persons and a floor area of 24.8 m^2^) from the European standard of EN16798-1 (Non-residential building; Category 1) [31]. The supply air was 100% outdoor air filtered by two-stage media filter (F6 and F9) and additional HEPA filter, so that background particle level was close to zero.

We studied four typical workplace layouts: Shared office 1 and 2 (without and with a common space), Meeting room, and Cafeteria. For instance, the Shared office 1 consisted of two or four office desks/chairs depending on the number of participants (two and four), and kettle and coffee machine on two cabinets (Fig. 1). The details of each floor plan with furniture organization are presented in Figure S1.

### Human participants

A total of six human participants were recruited (three males and females). The number of the participants was two and four for the two shared offices, and six for the Meeting room and Cafeteria. The selected occupancy number was based on occupancy density in office building specified by the Standard EN16798-1 [31]. The age of participants was between 26–31 and the average BMI ranged within 20.3–23.8 kg/m^2^ for females and 25.1–31.8 kg/m^2^ for males. We distributed the number of males and females equally in each experiment to minimize the impact of gender on human CO_2_ emission [11, 15] and maintained the same participants throughout the experiments. The participants wore typical office summer clothing (average 0.4 *clo*). One female participant (28 years old, BMI = 22.4 kg/m^2^) was designated as a reference participant for inhalation exposure measurements.

### Experimental design

We conducted a total of 11 chamber experiments during the summer period (13.07.2020–11.08.2020, Table S1). Each experiment was replicated two times except the cafeteria scenario. The measurements included the following three categories: air quality parameters (CO_2_, PM_2.5_, and PM_10_), contextual parameters (participants’ presence, number, body posture, and type of office activity) detected by PIRs and network cameras, and physiological parameters (skin temperature, heart rate and 3-axis acceleration) recorded by wearable wristbands. We determined seven sensor placements (IDs 1–7, Table 1) based on the literature and current best practices [24, 32, 33]. One example of monitor placement for the Shared office 1 is shown in Fig. 1, whereas the others are shown in Fig. S1. For breathing zone measurements, the reference participant wore one CO_2_ and one optical particle counter (OPC) at the sampling point located 20 cm below the nose (Fig. 1). The sampling tube connected to the CO_2_ monitor was fixed near the reference participant’s chest, whereas the OPC was placed in the pocket of an experimental vest. Two network cameras were installed at the ceiling and wall to provide ground-truth occupancy information.

**Table 1:** Monitor ID, measurement parameters, and placements.

ID	Parameters measured	Measurement placement (No. of monitors)	Measurement method
1	CO_2_, Size-resolved particle number concentration	**Front edge of participant desk (1)**	CO_2_ monitor, OPC
		*Front edge of desk near an abdomen of the reference participant*	
2		**Desk (1)**	CO_2_ monitor, OPC
		*On each participant’s desk*	
3		**Exhaust (2)**	CO_2_ monitor, OPC
		*Ceiling-mounted exhaust diffusers, 2.4* *m*	
		• *Exhaust 1 (E1*, Fig. 1*): near the reference participant*	
		• *Exhaust 2 (E2*, Fig. 1*): additional placement*	
4		**Breathing zone (1)**	CO_2_ monitor, OPC
		*20* *cm below from the reference participant’s nose*	
5	Participant presence, number, body posture, and type of office activity	**Ceiling (2)**	PIR, Network camera
		*Ceiling in the center of the chamber, 2.4* *m*	
6		**Wall (2)**	PIR, Network camera
		*Side wall, 1.4* *m and 2.0* *m*	
7	Participant presence	**Below the desk (1)**	PIR
		*Below the participant desk*	

The reference participant received wearable wristband before entering the chamber. Upon entering the chamber, the participants filled out the questionnaire about the seat number and their personal information (age, height, weight and clothing). During the experiment, the participants followed a set of scripted activities that were executed simultaneously by all. Seven activities were executed in two shared office spaces and six in the Meeting room and Cafeteria to simulate realistic occupancy interactions. All activities excluding entering and leaving the chamber were divided into two activity conditions: *sitting* activities and *standing* activities. *Standing* activities included standing or walking. A detailed description of scripted activities is provided in Fig. S2. Duration of each activity spanned from 5 to 25 min. All the participants exited the chamber after 60 min of the experiment and the chamber was sealed for 30 min to permit monitoring air pollutant concentration decay. The ethical and safety considerations of the experiments were approved by the Human Research Ethics Committee of EPFL.

### Research instrumentation

Two types of monitors were deployed to measure stationary indoor and breathing zone CO_2_ concentrations. Three HOBO MX CO_2_ Loggers (MX1102, Onset Computer Corporation, USA, measurement range: 0 to 5000 ppm, accuracy: ±50 ppm) were used for stationary indoor CO_2_ measurements. Additional two high-accuracy gas analyzers (LI-850, LI-COR Biosciences GmbH, Germany, measurement range: 0 to 20,000 ppm, accuracy: ±1.5%) with an air pump were deployed at the Exhaust 1 and at the Breathing zone of the reference participant. To capture size-resolved particle number concentration, we used four stationary and one wearable OPCs. Stationary monitors included: Met One 804 (Metone instruments, USA, 4 channels, size range: 0.3–10 µm, accuracy: ±10% to traceable standard) at the Exhaust 1 and the Front edge of participant desk; Met One HHPC 6 + (Beckman Coulter, USA, 6 channels, size range: 0.3–10 µm, counting efficiency: 50% at 0.3 µm (100% for particles >0.45 µm)) at the Exhaust 2; Mini-WRAS 1371 (GRIMM Aerosol Technik Ainring GmbH & Co., Germany, size range: 10 nm to 35 µm, >95% accuracy for single particle counting) on the Desk near the reference participant. One OPC (Met One 804) was worn by the reference participant.

Three PIR sensors (HOBO Occupancy/Light Data Logger, UX90-006x, Onset Computer Corporation, USA, Detection range: 12 m) were installed in the chamber. We also introduced one wearable wristband (E4, Empatica Inc., USA, frequency range: 32 Hz) that measured physiological state of the reference participant. Lastly, we installed two network cameras (M1065-LW and M3057-PLVE, Axis communications, Sweden, frequency range: 64 Hz) inside the chamber. All IAQ data were obtained at 1-min time intervals except for the CO_2_ measurements at breathing zone, which was measured at 0.5-s interval. The PIRs recorded occupancy information as binary code at 1-min time interval. Skin temperature was measured at 4 Hz frequency (0.25 s), heart rate at 1 Hz frequency (1 s) and acceleration at 32 Hz frequency (0.03125 s).

### Data analysis

Kierat et al. [16] proposed that accurate CO_2_ exposure assessment requires breathing zone measurements to be performed during the inhalation period only. To eliminate the effect of human exhalation, we selected only a single minimum value (Fig. S3) out of one respiratory cycle, where each cycle typically had six measurement points. Then the average breathing zone concentration was calculated as the average of the minimum concentrations recorded in each respiratory cycle. The possible lag between respiratory phase air sampling moment and the actual instrumental measurement time was removed. For breathing zone PM measurement, the full duration of the respiratory cycle was considered. The PM mass concentration (µg/m^3^) was estimated from measured number concentration by assuming that particles are spherical with density of 2.5 g/cm³, and by supposing that the mass-weighted size distribution, d*M*/d(log *d*_p_), is constant within each particle size group [34]. As density of indoor particles is typically in the range 1–2.5 g/cm³, the reported particle mass concentrations are likely to be upper-bound estimates [35].

We removed the contribution of the former activity to the CO_2_ and PM concentrations due to multiple participant activities conducted in a relatively short time period. We firstly estimated CO_2_ concentration by removing preceding 5-min average CO_2_ concentration from each time stamp (Fig. S4). For PM, we followed data processing approach described in [9] where the evolution of PM level from the former activity was calculated and removed (Fig. S5).

**Fig. 2:**
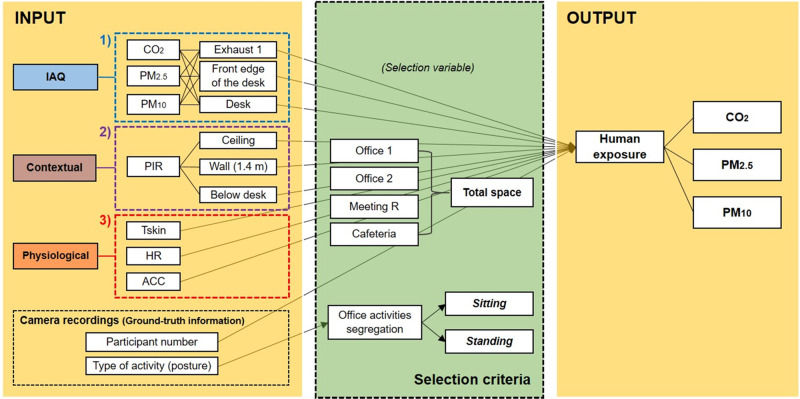
Input and output variables in composing MLR models. Selection criteria were applied while separating the collected data into *sitting* and *standing* activities. Notes: Exhaust 2 was not included as input in MLR analysis. Tskin stands for skin temperature, HR for heart rate, and ACC for resultant acceleration (${{{{{{{\mathrm{ACC}}}}}}}} = \sqrt {ACC\_x^2 + ACC\_y^2 + ACC\_z^2}$). Description of *sitting* and *standing* activities is shown in Figure S2.

Pearson correlation r value indicates existence of association between the measured variables, where stronger linear relationship appears as the r value approaches ±1 [36]. Our study examined *r* value between the measured IAQ parameters in order to identify the strength of the correlation between them. Through MLR analyses, we composed regression models by investigating the appropriateness of various physical parameters (presented as input variables) to estimate human exposure (presented as output variable) to CO_2_, PM_2.5_, and PM_10_ (Fig. 2). Firstly, we composed a regression model by using input variable from each data category: 1) air quality; 2) contextual; 3) physiological. We also included participant number as input variable to build a model that is not restricted to one specific office scenario. Data from all office layouts were integrated in analysis to create sufficient datasets to derive validate models. The ground-truth data (type of activity and body posture of the participants) acquired from network cameras allowed us to separate office activities into *sitting* and *standing*. Occupancy data obtained from PIR at Wall (2.0 m) and IAQ data of Exhaust 2 were excluded because of their limited datasets. Then, we composed regression models with input variables from all three different data categories and evaluated their accuracies compared to a model built with the air quality data only. The adjusted R^2^ values of each model were identified and compared to assess model accuracy, where the value of 0.75, 0.50, or 0.25 was deemed as strong, moderate or weak fit of the model as rule of thumb [37, 38]. Further, we examined *β* (standardized regression coefficients) to identify the positive or negative relationship between the input and output variables, and the magnitude of contribution of the input for estimation accuracy.

### Quality assurance

All the CO_2_ monitors and OPCs were calibrated ahead of the experiments with side-by-side test to eliminate the gap of measurement discrepancies among the monitors. The HOBO MX CO_2_ Loggers were inter-calibrated based on the linear correlation with the high-accuracy gas analyzer (LI-850) in a controlled chamber. The OPCs (two Met One 804 and one Met One HHPC 6+) were compared against the high-accuracy OPC (Mini-WRAS 1371). Adjustment factors of the side-by-side instrument performance tests are shown in Table S2.

## Results and discussions

### Summary of descriptive statistics and correlations of IAQ measurements

**Fig. 3:**
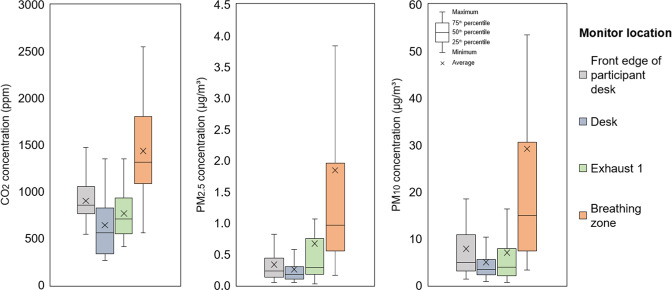
The CO_2_, PM_2.5_ and PM_10_ concentration at different stationary monitors across all activities and experiments.

In order to understand spatial IAQ variations in the chamber, we examined variations of studied air pollutant concentrations in relation to monitor placement. Figure 3 shows minimum, first quartile, median, third quartile, maximum and average CO_2_, PM_2.5_, and PM_10_ concentrations for each monitor placement (ID 1–4) averaged across all activities and experiments. Regardless of the air pollutant type, the breathing zone concentrations were substantially higher relative to stationary concentrations. The average of breathing zone CO_2_ concentrations of the reference participant were approximately two times higher than the ones from stationary monitors. This finding showed a notable increase in breathing zone CO_2_ concentration compared to a study by Melikov et al. [39], where CO_2_ concentration inhaled by a breathing thermal manikin was only 16% higher than in the room exhaust. The average PM_2.5_ and PM_10_ showed 6.7× and 6.8× higher concentrations at the breathing zone than the ones at stationary monitors, respectively.

The highest average CO_2_ and PM_10_ concentration among stationary IAQ monitors were recorded at the Front edge of participant desk which was the closest stationary monitor to the reference participant. This can be a result of exhaled CO_2_ jet that propagates downwards during *sitting* activities, as well as human thermal plume that transports locally generated airborne particles to the breathing zone [40]. This was not the case of PM_2.5_, where the highest average concentration among stationary monitors was detected at the Exhaust 1, likely because of vigorous activities (e.g., stuffing the cabinet with paper boxes) that occurred nearby. Further, we compared the absolute mean CO_2_ and PM_10_ concentration between the Exhaust 1 and Exhaust 2 (Fig. S6), where difference and variation of mean concentration were trivial in case of CO_2_, while it was significant in case of PM_10_.

**Fig. 4:**
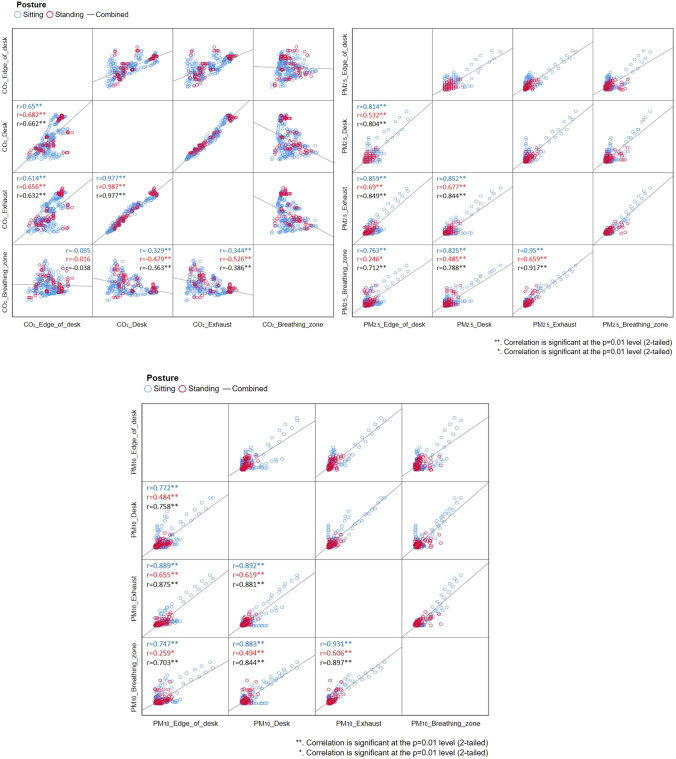
Pearson correlations of CO_2_, PM_2.5_, and PM_10_ measurements during *sitting*, *standing*, and *combined* participant activities.

Figure 4 shows the Pearson correlation *r* values between stationary indoor and breathing zone CO_2_ and PM concentrations during *sitting*, *standing*, and *combined* (*sitting* and *standing*) activities. Relative to *combined* activities, *r* values for CO_2_ were often higher when we segregated participant activity into *sitting* and *standing* activities. The correlation r between the CO_2_ in the breathing zone and at the Front edge of participant desk was 45% higher during *sitting* activities relative to *combined* activities. For *standing* activities, the relative increase was 36% and 32% at the Exhaust 1 and Desk locations compared to *combined* activities. CO_2_ measurements at the Exhaust 1 had a moderate correlation (*r* = 0.526) with the breathing zone measurements during *standing* activities. This finding agrees in part with a study by Pei et al. [24] who reported CO_2_ measured at the room exhaust well correlates with the inhalation exposure to CO_2_ under mixing ventilation. The two highest correlations between breathing and stationary CO_2_ measurement were at Exhaust 1 and Desk during *standing* activities. This is due to the contribution of spatial air pollution gradients and the proximity between the reference participant and the sensor locations during the *standing* activities. During the *sitting* activities, a relatively weak correlation (−0.3) between CO_2_ at the Exhaust 1 and in the Breathing zone may be attributed to spatial non-uniformity of air pollution concentration and greater distance between Exhaust 1 and seated reference participant. Lu et al. [41] also recognized that inconsistent patterns of CO_2_ concentrations in breathing zone of occupants may contribute to discrepancies of correlations between room exhaust and breathing zone CO_2_ level.

The correlation *r* between stationary and Breathing zone PM_2.5_ and PM_10_ measurement improved marginally during *sitting* activities (4–7%) and did not improve during *standing* activities compared to *combined* activities (Fig. 4). *Sitting* activities had better correlation for PM_2.5_ and PM_10_ than *standing* activities by threefold. Specifically, the correlation *r* between Exhaust 1 and Breathing zone during sitting condition showed over 0.9 for both PM_2.5_ and PM_10._ Low correlation between stationary and breathing zone PM levels during *standing* activities is attributed to irregular and high-intensity activities that resulted in highly episodic particle emissions. This result confirms that human inhalation exposure can be highly dependent on human activity and its intensity [17, 42]. Further, we compared correlation *r* between the two exhausts with the Breathing zone measurement (Table S3). In case of PM_10_, *r* value at Exhaust 2 decreased by 41–83% compared to the one at Exhaust 1 due to the distance between the reference participant and the deployed OPCs.

### Multiple linear regression models for estimating human exposure

#### MLR models based on stationary IAQ measurements

We investigated the accuracy of human exposure estimation to CO_2_, PM_2.5_, and PM_10_ by using the input variables from the stationary IAQ monitors. Regression model for each studied air pollutant was proposed while considering a different number (1, 2, or 3) and combination of IAQ input variables. Table 2 shows adjusted *R*² values of each model under *combined* and separated *sitting* and *standing* activities. Segregated human activities can improve inhalation exposure estimation for all studied air pollutants. During *standing* activities, accuracy for estimating CO_2_ inhalation exposure was 77% higher compared to one under *combined* activities. This result agrees with the previous report (Fig. 4) of significant improvement of correlation between stationary and breathing zone CO_2_ measurements when participants’ activities were separated. Accuracy of PM_2.5_ and PM_10_ exposure estimation was 8% higher during *sitting* activities (adjusted *R*^2^ 0.93 and 0.91 respectively) compared to the ones during *combined* activities. In case of PM, *sitting* activities had better estimation accuracy relative to *combined* activities owing to a closer distance between seated participants and the OPCs with a fewer episodic particle emission relative to *standing* activities. Licina et al. [42] also identified personal cloud effect with elevated PM concentration in breathing zone of seated occupants while reporting that well-mixed representation of indoor space might underestimate human exposure to coarse particles. During *sitting* activities, the best single input variable for PM_2.5_ and PM_10_ exposure detection was PM measurement at the Exhaust 1 (*R*^2^ of 0.91 and 0.87), which was located near the head of the reference participant.

**Table 2:** Adjusted *R*² value of MLR models for IAQ exposure estimation by using different numbers and combinations of stationary CO_2_ and PM measurements during *combined* and separated activities (*sitting* and *standing*).

Number of variables	IAQ stationary monitor placement	*Combined* activities^a^			*Sitting*^a^			*Standing*^a^		
		CO_2_	PM_2.5_	PM_10_	CO_2_	PM_2.5_	PM_10_	CO_2_	PM_2.5_	PM_10_
1	Front edge of participant desk	0.326	**0.516**	0.495	0.26	**0.61**	**0.58**	**0.579**	0.068	0.073
	Desk	0.292	**0.671**	**0.731**	0.24	**0.77**	**0.82**	**0.517**	0.215	0.224
	Exhaust 1	0.291	**0.841**	**0.803**	0.24	**0.91**	**0.87**	**0.514**	0.442	0.363
2	Front edge of participant desk + Desk	0.328	**0.68**	**0.738**	0.26	**0.78**	**0.83**	**0.581**	0.202	0.214
	Front edge of participant desk + Exhaust 1	0.328	**0.855**	**0.831**	0.26	**0.92**	**0.90**	**0.584**	**0.501**	0.376
	Desk + Exhaust 1	0.29	**0.843**	**0.819**	0.23	**0.91**	**0.89**	**0.512**	0.433	0.371
3	Front edge of participant desk + Desk + Exhaust 1	0.326	**0.861**	**0.842**	0.26	**0.93**	**0.91**	**0.578**	0.498	0.396

Bolded values have moderate or strong correlation (*R*^2^ > 0.5).

^a^All models included participant number as one input variable

The CO_2_ exposure estimation by using a single stationary IAQ monitor during *sitting* activities was not accurate (average adjusted *R*² = 0.25 across all single monitors, Table 2). Furthermore, the PM_2.5_ and PM_10_ exposure estimations by using a single OPC during *standing* activities were also not accurate (average adjusted *R*² of 0.24 and 0.22 across all single OPCs, Table 2). The results indicate that the single stationary IAQ monitoring location recommended by standards and guidelines [19, 20, 43] does not capture exposure well and the measurements may not be reliable particularly when complex airflow interactions exist in the space.

Using all three IAQ inputs (Front edge of participant desk + Desk + Exhaust 1) for estimating PM_2.5_ and PM_10_ exposure showed 2% and 5% higher adjusted *R*^2^ for *sitting* activities, and 13% and 9% higher adjusted *R*^2^ for *standing* activities relative to using single IAQ input. This was not the case for CO_2_ exposure estimation, where there was no difference between using single and multiple variables. Further, we reported regression coefficients of the models (Table S4) consisted of a single stationary IAQ measurement and participant number as input variables with the best estimation accuracy. The regression equations (Eqs. 1–3) are listed based on the models (Table S4) composed with one stationary IAQ measurement and participant number (*part*_*num*_) as inputs. A negative correlation between participant number and CO_2_ inhalation exposure was observed, while a positive correlation between CO_2_ level at the Front edge of participant desk and CO_2_ inhalation exposure was detected during *standing* activities (Eq. 1). As indicated in Eq. (2) and Eq. (3), two inputs (*part*_*num*_, *part*_*exhaust*_) had a positive correlation with output (inhalation exposure to PM_2.5_ and PM_10_) during *sitting* activities. Interestingly, inhalation exposure to PM_10_ was more dependent on the participant number than the stationary PM_10_ measurement at the ventilation exhaust, while the opposite aspect was shown for inhalation exposure to PM_2.5_.

1 $$CO_{2,\,exposure} =	 - 281.51part_{num} + 0.829CO_{2,\,front\,edge\,of\,participant\,desk}\\ 	+ 1983.328$$

2 $$PM_{2.5,exposure} = 0.172part_{num} + 1.795PM_{2.5,\,exhaust}-0.007$$

3 $$PM_{10,\,exposure} = 2.497part_{num} + 1.652PM_{10,\,exhaust} + 1.098$$

#### MLR models based on contextual measurements

We derived the MLR models by using input variables obtained from PIRs installed at three different placements; ceiling, wall, and below the participant desk. Table 3 summarizes adjusted *R*² values of each model with different combination of inputs under *combined* and separated *sitting* and *standing* activities. The estimation accuracy did not show any significant *R*² values throughout all proposed models, meaning that the human presence/absence data is generally not effective in detecting personal exposures. However, data obtained by all three PIRs was moderately effective (*R*² > 0.5) in estimating inhalation exposure to CO_2_ during *standing* activities. Our results point towards conclusion that the PIR alone is able to detect human presence in the space (see *β* = 0.26, Table S5), but none of the three PIRs showed a sufficient ability to estimate inhalation exposure solely.

**Table 3:** Adjusted *R*² value of MLR models for IAQ exposure estimation by using different combinations of PIRs measurements during *combined*, *sitting*, and *standing* activities.

Number of variables	PIR measurement placements	*Combined* activities			*Sitting*			*Standing*		
		CO_2_	PM_2.5_	PM_10_	CO_2_	PM_2.5_	PM_10_	CO_2_	PM_2.5_	PM_10_
1	Ceiling	0.294	0.004	0.003	0.241	0.008	0.007	**0.505**	−0.006	−0.004
	Wall	0.288	−0.006	−0.006	0.247	0.002	0.002	**0.568**	0.002	−0.01
	Below desk	0.296	0.011	0.017	0.247	0.019	0.026	**0.526**	−0.012	−0.016
2	Ceiling + Wall	0.292	0.000	0.000	0.25	0.005	0.005	**0.561**	−0.005	−0.01
	Ceiling + Below desk	0.299	0.017	0.022	0.25	0.023	0.03	**0.518**	−0.022	−0.02
	Wall + Below desk	0.297	0.01	0.015	0.277	0.015	0.023	**0.57**	−0.009	−0.021
3	Ceiling + Wall + Below desk	0.301	0.016	0.021	0.279	0.02	0.026	**0.563**	−0.018	−0.024

Bolded values have moderate correlation (*R*^2^ > 0.5).

#### MLR models based on physiological measurements

We also examined MLR models composed of physiological measurements from wearable wristband (E4), which included the skin temperature (*T*_*skin*_), heart rate (*HR*), and resultant three-axis acceleration (*ACC*) of the reference participant. Adjusted R² values of each model under *combined*, *sitting* and *standing* activities are presented in Table 4. In general, physiological measurements gave poor estimate of inhalation exposures for the investigated scenarios except the CO_2_ exposure in *standing* activities that had a moderate accuracy (*R*² > 0.5). A discrepancy of estimation accuracy between *sitting* and *standing* activities is aligned with the findings of two experimental studies [44, 45] that indicated a complex relationship of human physiological status and indoor CO_2_ concentration. Having more than one physiological parameter could improve the estimation accuracy relative to single measurement in some cases. For example, the model accuracy for detecting PM_2.5_ and PM_10_ exposure by multiple inputs showed 5 and 10% increase in sitting activities and showed 10% increase in standing activities in case of CO_2_ compared to the model with a single input. However, overall model accuracy by physiological inputs was still insufficient to estimate inhalation exposures. Further, we reported regression coefficients of a model that best estimated CO_2_ exposure (adjusted *R*² = 0.594) by physiological inputs, where large β coefficient was shown in order of participant number, *T*_*skin*_, and *HR* (Table S6).

**Table 4:** Adjusted R² value of MLR models for IAQ exposure estimation by using different combinations of wearable wristband measurements during *combined*, *sitting*, and *standing* activities.

Number of variables	Wearable wristband parameters^a^	*Combined* activities			*Sitting*			*Standing*		
		CO_2_	PM_2.5_	PM_10_	CO_2_	PM_2.5_	PM_10_	CO_2_	PM_2.5_	PM_10_
1	Tskin	0.407	0.039	0.016	0.477	0.121	0.067	**0.528**	−0.015	−0.017
	HR	0.3	−0.006	−0.006	0.237	0.001	0.000	**0.54**	−0.007	−0.003
	ACC	0.288	−0.003	−0.002	0.235	0.005	0.005	**0.506**	−0.014	−0.017
2	Tskin + HR	0.459	0.04	0.014	0.475	0.118	0.062	**0.594**	−0.022	−0.019
	Tskin + ACC	0.405	0.043	0.021	0.476	0.13	0.074	**0.521**	−0.031	−0.031
	HR + ACC	0.3	−0.006	−0.004	0.234	0.001	0.002	**0.537**	−0.022	−0.016
3	Tskin + HR + ACC	0.457	0.043	0.018	0.474	0.127	0.07	**0.589**	−0.038	−0.032

Bolded values have moderate correlation (*R*^2^ > 0.5).

^a^*Tskin* skin temperature, *HR* heart rate, ACC resultant acceleration ($\sqrt {ACC\_x^2 + ACC\_y^2 + ACC\_z^2}$)

#### MLR models based on multiple parameter measurements

We finally derived MLR models by combining stationary IAQ, physiological (E4) and contextual (PIR) parameters and compared the results with the models composed of a single parameter. We examined the models under segregated activities (*sitting* and *standing*), which was more advantageous in terms of model accuracy relative to *combined* activities as previously noted in “MLR models based on stationary IAQ measurements”. Adjusted R² values of each model were reported with relevant input variables listed in parentheses (Table 5). In case of *sitting* activities, the estimation accuracy showed twofold (101%) increase by using multiple parameters (IAQ+E4+PIR) compared to the model with a single stationary CO_2_ measurement. When participants were moving around, CO_2_ exposure estimation was better by integrating stationary CO_2_ measurements with wearable (*T*_*skin*_, *HR*) and PIR (*PIR*_*Wall*) measurement, however, the improvement was small (4–6% increase).

**Table 5:** Adjusted *R*² value (relevant input variables) of MLR models with combined input parameters for IAQ exposure estimation during *sitting* and *standing* activities.

Combinations of parameters^a^ *(used as input variables)*	Adjusted *R*² of composed MLR model (relevant input variables^b^)					
	*Sitting*			*Standing*		
	CO_2_ estimation	PM_2.5_ estimation	PM_10_ estimation	CO_2_ estimation	PM_2.5_ estimation	PM_10_ estimation
**Single IAQ**	0.26 (Part_num, CO_2__Front edge of participant desk)	0.91 (Part_num, PM_2.5__Exhaust 1)	0.87 (Part_num, PM_10__Exhaust 1)	0.579 (Part_num, CO_2__Front edge of participant desk)	0.442 (Part_num, PM_2.5__Exhaust 1)	**0.363** **(Part_num, PM**_**10**_**_ Exhaust 1)**
**IAQ + E4**	0.492 (Part_num, CO_2__Desk, Exhaust 1, Tskin)	0.931 (Part_num, PM_2.5__ Front edge of participant desk, Desk, Exhaust 1, HR)	**0.925** **(Part_num, PM**_**10**_**_ Front edge of participant desk, Desk, Exhaust 1, Tskin, HR)**	0.594 (Part_num, Tskin, HR)	**0.503** **(PM**_**2.5**_**_ Front edge of participant desk, Exhaust 1)**	0.363 (Part_num, PM_10__ Exhaust 1)
**IAQ + PIRs**	0.292 (Part_num, CO_2__ Front edge of participant desk, PIR_Wall, Desk)	0.933 (Part_num, PM_2.5__ Front edge of participant desk, Desk, Exhaust 1, PIR_Wall, Desk)	0.912 (Part_num, PM_10__ Front edge of participant desk, Desk, Exhaust 1, PIR_ceiling)	**0.615** **(Part_num, CO**_**2**_**_ Front edge of participant desk, PIR_Wall)**	0.503 (PM_2.5__ Front edge of participant desk, Exhaust 1)	0.363 (Part_num, PM_10__ Exhaust 1)
**IAQ + E4 + PIRs**	**0.524** **(Part_num, CO**_**2**_**_Desk, Exhaust 1, Tskin, PIR_Wall, Desk)**	**0.939** **(Part_num, PM**_**2.5**_**_ Front edge of participant desk, Desk, Exhaust 1, HR, PIR_Wall, Desk)**	0.925 (Part_num, PM_10__ Front edge of participant desk, Desk, Exhaust 1, Tskin, HR)	0.594 (Part_num, Tskin, HR)	0.503 (PM_2.5__ Front edge of participant desk, Exhaust 1)	0.363 (Part_num, PM_10__ Exhaust 1)
**Improvement of estimation accuracy** *(Single IAQ vs combination of parameters, percent increase %)*	101	3.2	6.3	6.2	13.8	0

Bolded characters in parentheses indicate the best parameter or a combination of parameters to estimate inhalation exposure. The last row of the table indicates how much percent increase (%) was obtained in terms of estimation accuracy when using combined parameters compare to using a single IAQ parameter.

^a^IAQ: IAQ measurement from stationary IAQ monitors, E4: Physiological measurement from wearable sensor, and PIRs: Contextual measurement from PIR sensor

^b^*Part_num* number of participants, *Tskin* skin temperature, *HR* heart rate

The relevant inputs for PM_2.5_ and PM_10_ estimation during *standing* activities were stationary PM measurements but did not include any contextual or physiological indicators. During *sitting* activities, however, physiological state (*T*_*skin*_, *HR*) of the participant was included as relevant input for PM exposure detection. Particularly, the skin temperature (*T*_*skin*_) was advantageous in estimating PM_10_ exposure while heart rate (*HR*) was useful in estimating both PM_2.5_ and PM_10_ exposures. By combining IAQ with wearable and PIR measurements, adjusted R² for PM_2.5_ and PM_10_ exposure estimation models slightly improved (3–6% increase in *sitting* activities). During *standing* activities, having two stationary monitors increased the estimation accuracy by 14% compared to having a single OPC monitor. This increase, however, has little relevance as the single IAQ input was sufficient to accurately estimate the exposure.

Except a notable improvement (twofold increase) of using combined parameters in CO_2_ exposure estimation, the increase of model accuracy by combining the parameters was trivial. The regression equations of the best models with combined input parameters are reported as Eqs. (S1–S5). We also included normality test of the final regression models (Fig. S7) in order to make valid future inferences of the models. Lastly, we presented additional regression models that used single and combined parameters during *combined* (*sitting* + *standing*) activities (Table S7). As expected, the best model accuracy for estimating personal exposure to CO_2_, PM_2.5_ and PM_10_ was not apparent when participants’ activities were mixed. This finding confirms the importance of having contextual information, particularly occupant activities, for evaluating personal exposures.

### Study limitations

Our study has several limitations. Firstly, our findings are limited to a handful of selections of office setups, activities, single air change rate, and single room air distribution strategy, which means our propositions may not be applicable to completely different circumstances. Our models might have been different if the exhaust vent was not positioned near the seated reference participant, as evidenced by analyzing indoor air pollution and correlation with breathing zone concentration between two different placements of exhaust (Exhaust 1 and 2). Furthermore, being limited to measuring personal exposure of one participant, we cannot generalize expiratory characteristics (e.g., the geometry of a person’s nose, lung capacity, the position of a head) to all population. Physical intrusiveness of measurements to the participants remains a weakness because it could have influenced their movements. Lastly, experimental instruments were worn by the reference participant with a real-time camera recording, which would not be possible in a real-life scenario due to intrusiveness and privacy issues [46, 47]. To tackle these limitations, one promising technology is a novel camera-based human activity detector algorithm named PifPaf [48] that gives information about total number of participants and estimates the posture of participants containing 17 joints, without violating privacy issues.

## Conclusion

Considering the challenges of direct measurements of human inhalation exposures, it is useful to explore the effectiveness of alternative methods for approximating exposure to typical indoor air pollutants. In a ventilated chamber with dynamic occupancy, we deployed three different sensing techniques (stationary IAQ, contextual and physiological measurements) to detect breathing zone CO_2_, PM_2.5_, and PM_10_ concentrations.

The accuracy of estimating inhalation exposures was contingent upon occupant number, activities, and positioning of sensors. Firstly, occupant number was relevant in estimating exposures to investigated air pollutants except the case of PM_2.5_ in *standing* activities. A clear improvement of estimation accuracy was observed by segregating data into *sitting* and *standing* activities; the relative improvement was 9–60% during *sitting* compared to *combined* activities. Vigorous *standing* activities had higher correlation between stationary and breathing zone CO_2_ measurement, attributed to reduced spatial air pollution gradients in the chamber. On the contrary, dynamic activities resulted in reduced correlation between stationary and breathing zone PM measurements due to the highly episodic and localized emissions. The CO_2_ and PM measurement at ceiling-mounted ventilation exhaust above the reference participant showed the highest correlation with the breathing zone measurement regardless of activities.

Through regression analyses, the best IAQ sensor placement for personal exposure estimation was the Front edge of participant desk for CO_2_ and the ventilation exhaust for PM. Specifically, the Front edge of the desk showed a moderate accuracy (adjusted *R*^2^ = 0.58) for CO_2_ inhalation exposure estimation of a standing participant. The PM measurements at the exhaust showed the substantial potential (adjusted *R*² > 0.8) as a proxy to detect personal exposure to PM_2.5_ and PM_10_ of a seated participant. By combining multiple inputs (environmental, physiological, and contextual parameters), the model estimation on inhalation exposure to CO_2_ improved by twofold during *sitting* activities, while the improvement was limited in case of PM (~10%). Our findings indicate that the personal exposure estimation could be enhanced by possessing contextual information (e.g., body posture and type of activity), although the improvement can be trivial in specific cases.

This study contributes to broadening the knowledge of proxy methods for detecting personal air pollution exposures under dynamic occupancies, which goes beyond the existing investigations typically performed under the static conditions [15, 16, 42]. Our findings are novel since it involves contextual and physiological parameters in the actual exposure estimation compared to the previous studies that only investigated the correlation between room occupancy information and exposures [26–28].

The practical recommendations on optimal monitor placement indoors could help stakeholders better understand a real human exposure to air pollutants and secure good IAQ in buildings. Placing a single IAQ monitor at a proper location can be a practical solution while minimizing the initial cost of monitor purchase and its maintenance fee. However, combined monitoring strategies (environmental, physiological, and contextual) could reduce potential errors resulting from having one monitor installed at suboptimal location. Further investigations should generalize the regression models under different space contexts. Future developments of automatic occupancy detections are needed to develop a more robust and cost-effective approach for human exposure detection and management.

## Supplementary Information

SUPPLEMENTARY INFORMATION

## Data Availability

All data generated or analyzed during this study are included in this published article and its supplementary information files.
