# Supplementary material for: Proxy methods for detection of inhalation exposure in simulated office environments
Source: J Expo Sci Environ Epidemiol. 2022 Nov 8;33(3):396–406. doi: 10.1038/s41370-022-00495-w (PMC10234809; doi:10.1038/s41370-022-00495-w)
Supplement: Supplementary file 1 — SUPPLEMENTARY INFORMATION [file 41370_2022_495_MOESM1_ESM.docx]

SUPPLEMENTARY INFORMATION

**Proxy methods for detection of inhalation exposure in simulated office environments**

Seoyeon Yun^1*^, Sailin Zhong^2^, Hamed S. Alavi^3^, Alexandre Alahi^4^, Dusan Licina^1^

^1^ Human-Oriented Built Environment Lab, School of Architecture, Civil and Environmental Engineering, École Polytechnique Fedérale de Lausanne, Switzerland
^2^ Human-IST Institute, Department of Informatics, University of Fribourg, Switzerland

^3^ Digital Interactions Lab, Institute of Informatics, University of Amsterdam, Netherlands
^4^ Visual Intelligence for Transportation, School of Architecture, Civil and Environmental Engineering, École Polytechnique Fedérale de Lausanne, Switzerland

*^*^Corresponding email:* [*seoyeon.yun@epfl.ch*](mailto:seoyeon.yun@epfl.ch)


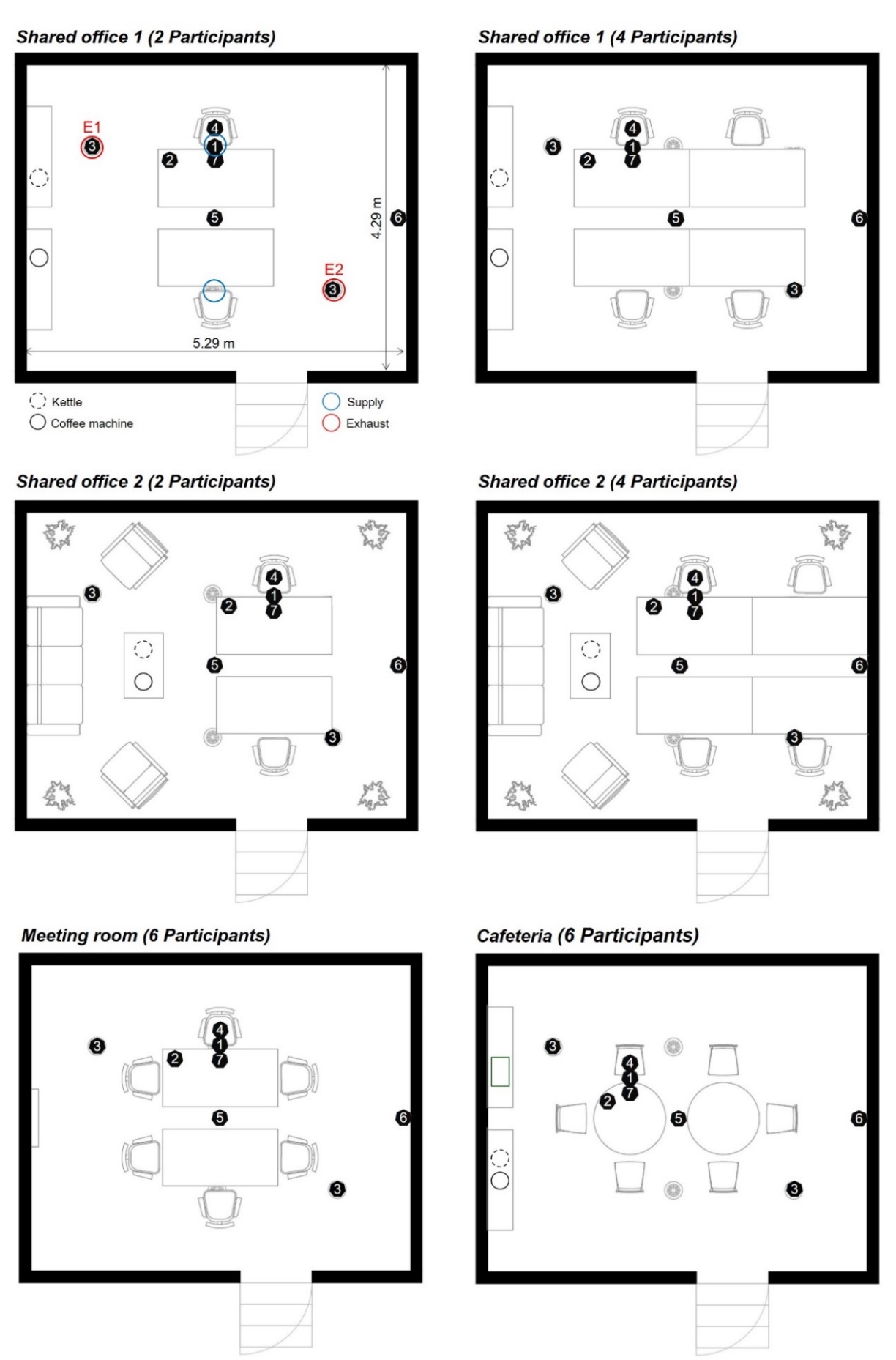


**Figure S1.** Floor plan and monitor placement IDs (1 -7). The dimension of the space and supply/exhaust diffuser placement are all the same in every space as shown in the Shared office 1 (2 Participants). The Shared office 1 consisted of two or four office desks/chairs depending on the number of participants (two and four), and kettle and coffee machine on two cabinets. In Shared office 2, the office desk/chair setup was similar to Shared office 1 but it had a common space where the participants could sit on fabric sofa and have coffee/tea from a table. The Meeting room (six participants) was equipped with two desks with six office chairs and TV screen to simulate actual group meeting activity. The Cafeteria (six participants) was composed of two lounge tables in the middle of the space with six chairs with two cabinets to place coffee machine, kettle, and microwave.

**(i) Activity scenario for Shared office 1 (w/o common space)**

**(ii) Activity scenario for Shared office 2 (w/ common space)**

**(iii) Activity scenario for Meeting room**

**(iv) Activity scenario for Cafeteria**

**Figure S2**. Participants’ office activities (duration in minutes) in each space type. *Sitting* activities are marked as blue shading while *standing* activities are marked as orange shading. “Entering”, “Leaving” and “One-person standing/presenting” activity were excluded in regression analyses.


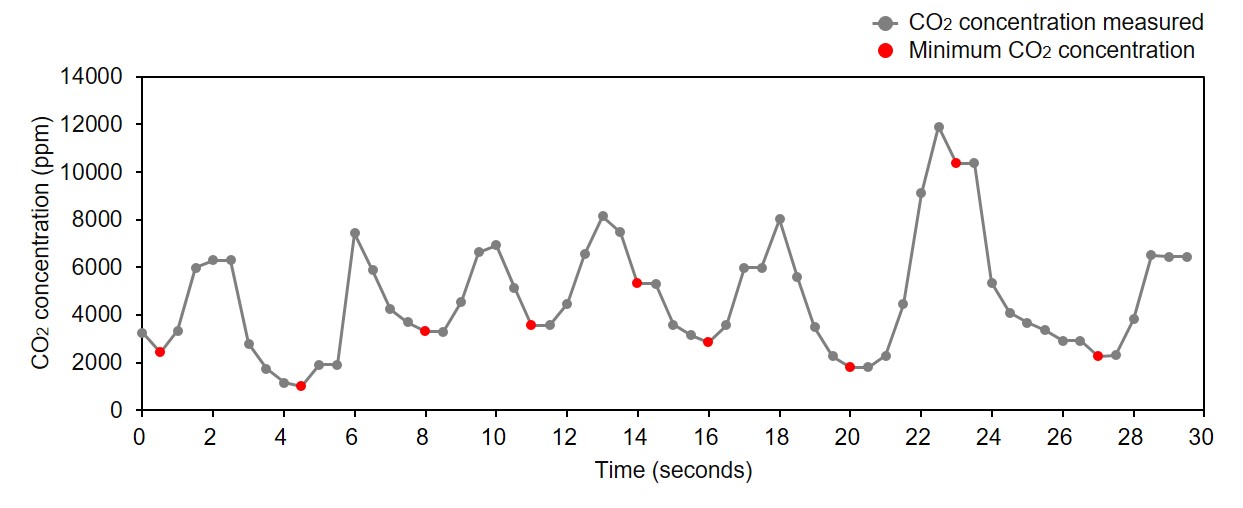


**Figure S3.** Calculation of inhaled CO_2_ concentration during 30-second breathing cycle of the reference participant. The red dots were taken into account for calculating inhaled CO_2_ concentration.


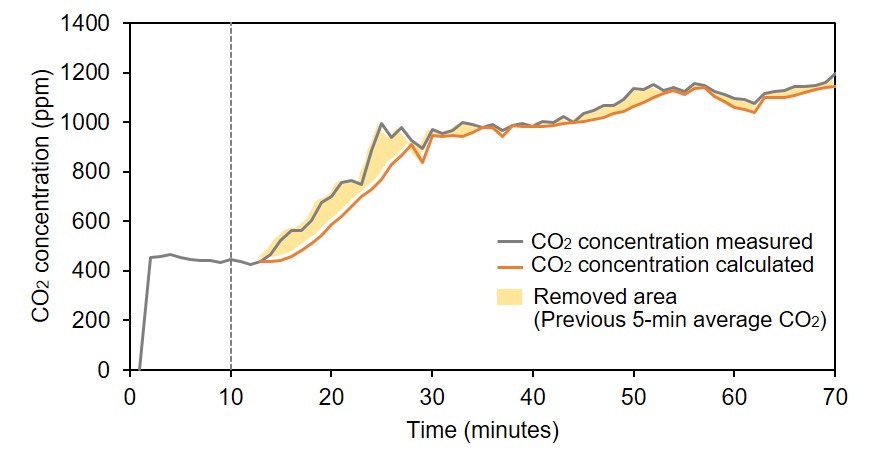


**Figure S4**. Calculated CO_2_ concentration based on subtracting former 5-minute CO_2_ concentration from each time stamp. (e.g. calculated CO_2_ concentration at 40 minutes: measured CO_2_ concentration at 40 minutes – average measured CO_2_ concentration from 35 to 39 minutes)

**
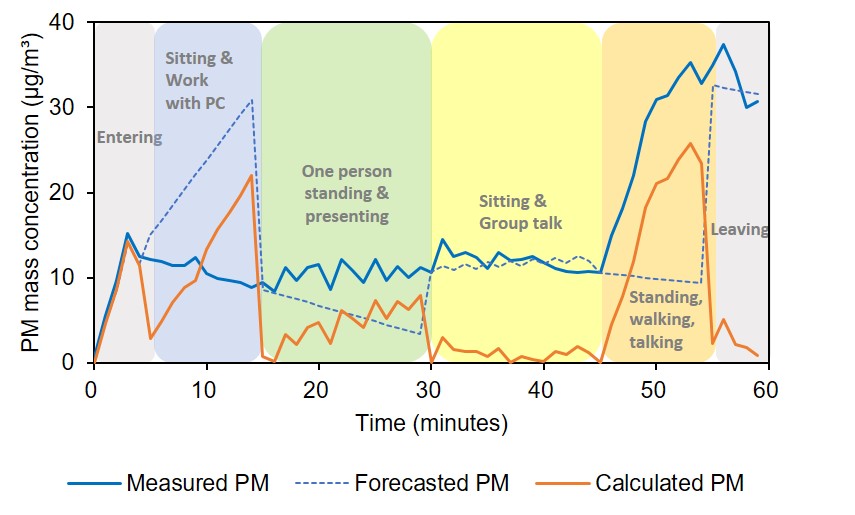
**

**Figure S5**. Calculated PM mass concentration for each participant activity by using forecast curve based on the data of the former activity and removing the gap between measured and predicted PM value from the measured PM value. Forcasted PM has been processed using the FORECAST.ETS function (target_date, values, timeline) in Excel, where the contribution of forecasted PM has been removed from measured PM so that we could minimize the contribution of former activity on the latter PM value from another activity.


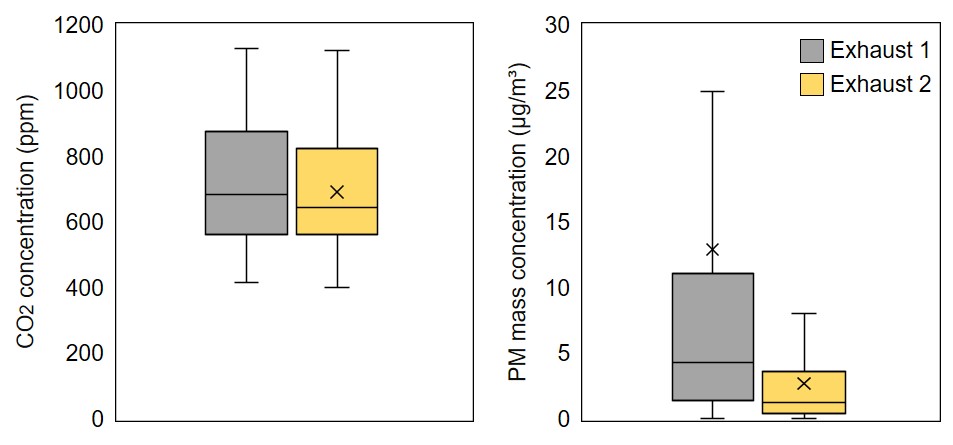


**Figure S6**. Comparison of mean CO_2_ and PM_10_ concentration at two exhausts for *combined* activities (*sitting* and *standing*) in the space. The higher PM_10_ concentration at the Exhaust 1 compared to the Exhaust 2 was likely attributed to vigorous activities (i.e., stuffing the cabinet with paper boxes) that occurred near the Exhaust 1.

**(i)** **Normal P-P plot for CO_2_ exposure estimation model (left: *sitting activities*, right: *standing* activities)**


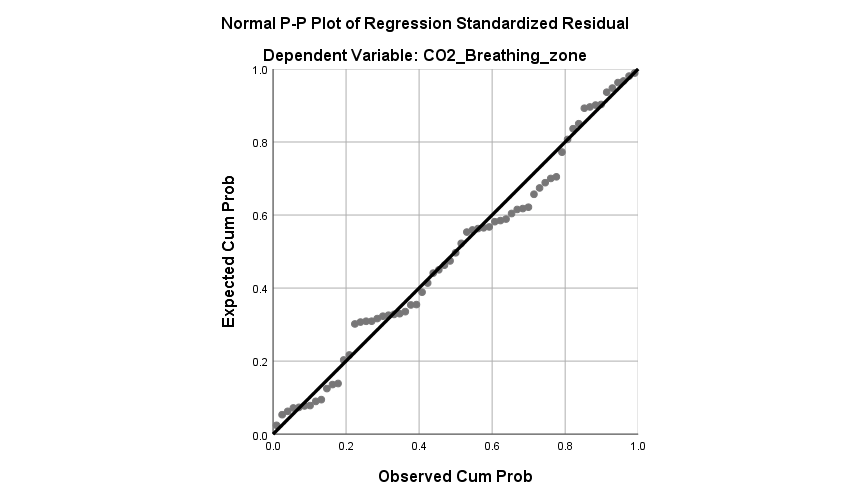

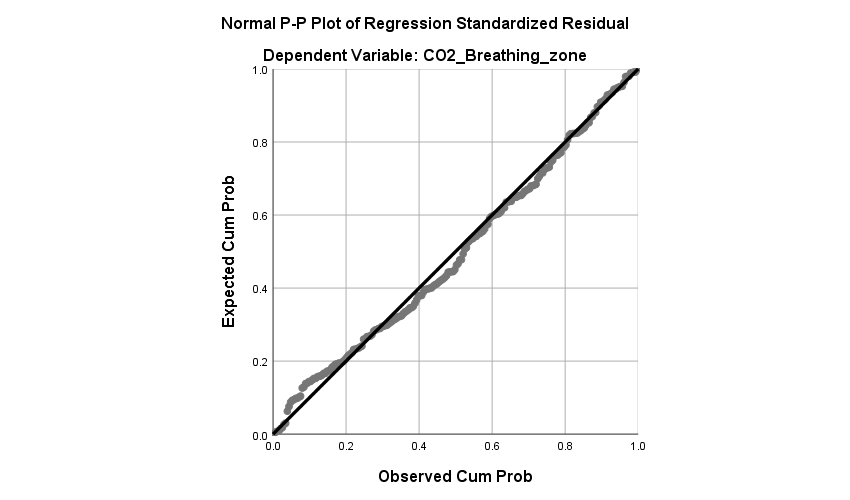


**(ii)** **Normal P-P plot for PM_2.5_ exposure estimation model (left: *sitting activities*, right: *standing* activities)**


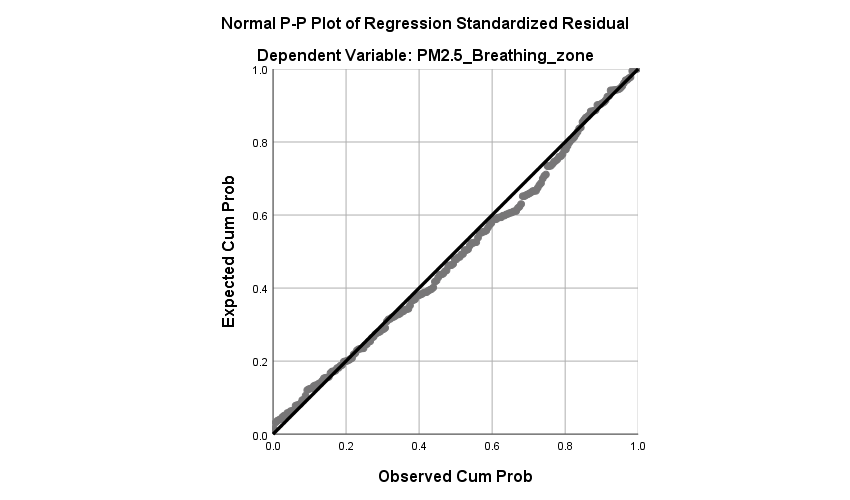

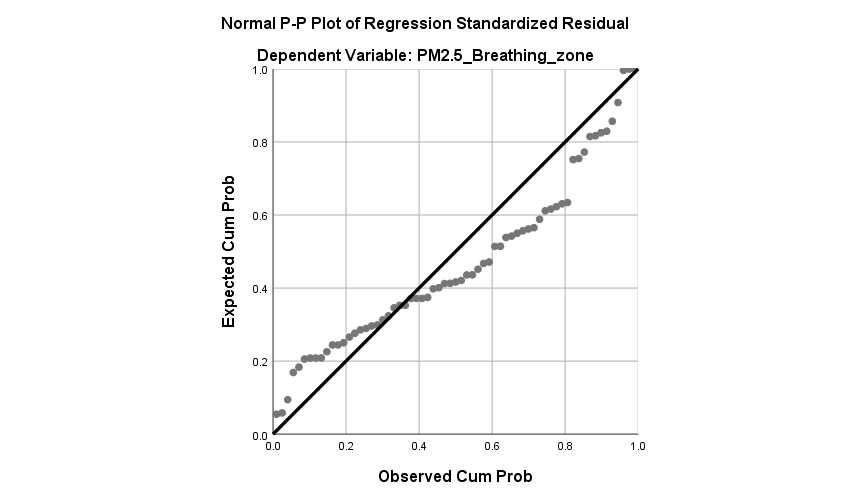


**(iii)** **Normal P-P plot for PM_10_ exposure estimation model (*sitting* activities)**


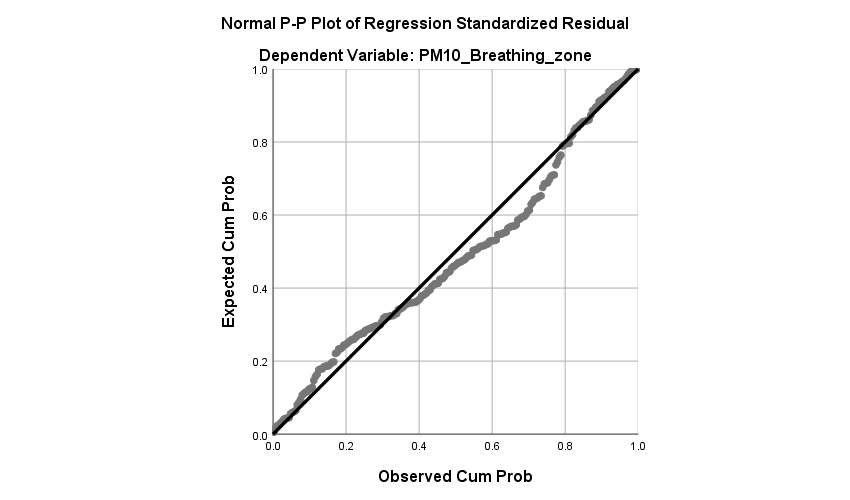


**Figure S7**. Normal P-P plot of composed regression models: test for normal distribution of residuals. Test for PM10 exposure estimation model in *standing* activities was excluded.

**Table S1.** List of experimental runs, associated occupancy and environmental conditions.

| Experimental ID | Space type | No. of participants | ACH | T, RH |
| --- | --- | --- | --- | --- |
| 1 | Shared office 1 | 2 | 2.4 - 2.6 h^-1^ | 24.9±0.4 ℃, 54.3±4 % |
| 2 |  | 2 |  |  |
| 3 |  | 4 |  |  |
| 4 |  | 4 |  |  |
| 5 | Shared office 2 | 2 |  |  |
| 6 |  | 2 |  |  |
| 7 |  | 4 |  |  |
| 8 |  | 4 |  |  |
| 9 | Meeting room | 6 |  |  |
| 10 |  | 6 |  |  |
| 11 | Cafeteria | 6 |  |  |

**Table S2.** Adjustment factors to mutually correct IAQ instruments. Reference instrument is shown in the bracket.

| Monitor placement | | | Adjustment factors | | |
| --- | --- | --- | --- | --- | --- |
| ID | Description | CO_2_ | | PM | |
|  |  |  |  | PM_2.5_ | PM_10_ |
| 1 | Front edge of participant desk | 1.177 | | 0.8565 | 1.618 |
| 2 | Desk | 1.124 | | 1 (reference, Mini-WRAS 1371) | |
| 3 | Exhaust 1 | 1 (reference, LI-850) | | 1.1021 | 1.4971 |
| 4 | Breathing zone | 0.986 | | 1.2435 | 1.6801 |

**Table S3.** Correlation r comparison between the two Exhausts and the Breathing zone.

| Stationary monitor  location | *Sitting* activities | | *Standing* activities | |
| --- | --- | --- | --- | --- |
|  | Breathing zone CO_2_ | Breathing zone PM_10_ | Breathing zone CO_2_ | Breathing zone PM_10_ |
| Exhaust 1 | -0.344** | 0.931** | -0.526** | 0.606** |
| Exhaust 2 | -0.517** | 0.551** | -0.491** | -0.106 |
| Difference  (Exhaust 2 compared to Exhaust 1, percent increase %) | 50.3 % | -40.8 % | -6.7 % | -82.5 % |

**. Correlation is significant at the p=0.01 level (2-tailed)

**Table S4.** Regression coefficients for estimating CO_2_, PM_2.5_, and PM_10_ exposure using one stationary IAQ monitor and participant number. Notes: B stands for unstandardized regression coefficient, Std. Error for unstandardized standard error of the B, β for standardized regression coefficient, t for t-value, and p for p-value.

| **Model coefficients^a,b,c^** | | | | | |
| --- | --- | --- | --- | --- | --- |
| Variable | B | Std. Error | β | t | p |
| (Constant) | 1983.328 | 214.483 |  | 9.247 | 0.000 |
| Participant_number | -281.51 | 29.675 | -0.828 | -9.486 | 0.000 |
| CO_2__ Front edge of participant desk | 0.829 | 0.25 | 0.289 | 3.316 | 0.002 |

^a.^ Dependent variable: CO_2__Breathing_zone

^b.^ R²_adj_ = 0.579 (N = 65, p = 0.000)

^c.^ CO_2_ exposure estimation model for *standing* activities

| **Model coefficients^a,b,c^** | | | | | |
| --- | --- | --- | --- | --- | --- |
| Variable | B | Std. Error | β | t | p |
| (Constant) | -0.007 | 0.182 |  | -0.037 | 0.971 |
| Participant_number | 0.172 | 0.043 | 0.081 | 3.954 | 0.000 |
| PM_2.5__Exhaust 1 | 1.795 | 0.039 | 0.949 | 46.319 | 0.000 |

^a.^ Dependent variable: PM_2.5__Breathing_zone

^b.^ R²_adj_ = 0.91 (N = 220, p = 0.000)

^c.^ PM_2.5_ exposure estimation model for *sitting* activities

| **Model coefficients^a,b,c^** | | | | | |
| --- | --- | --- | --- | --- | --- |
| Variable | B | Std. Error | β | t | p |
| (Constant) | 1.098 | 3.482 |  | 0.315 | 0.753 |
| Participant_number | 2.497 | 0.833 | 0.073 | 2.997 | 0.003 |
| PM_10__Exhaust 1 | 1.652 | 0.043 | 0.929 | 38.294 | 0.000 |

^a.^ Dependent variable: PM_10__Breathing_zone

^b.^ R²_adj_ = 0.91 (N = 220, p = 0.000)

^c.^ PM_10_ exposure estimation model for *sitting* activities

**Table S5.** Regression coefficients for estimating CO_2_ exposure using one input [PIR_Wall (1.4 m)] from PIRs and participant number under *standing* activities.

| **Model coefficients^a,b^** | | | | | |
| --- | --- | --- | --- | --- | --- |
| Variable | B | Std. Error | β | t | p |
| (Constant) | 1842.901 | 270.202 |  | 6.82 | 0.000 |
| Participant_number | -265.276 | 28.731 | -0.78 | -9.233 | 0.000 |
| PIR_Wall (1.4 m) | 831.186 | 275.675 | 0.255 | 3.015 | 0.004 |

^a.^ Dependent variable: CO_2__Breathing_zone

^b.^ R²_adj_ = 0.568 (N = 65, p = 0.000)

**Table S6.** Regression coefficients for estimating CO_2_ exposure using two inputs [Tskin + HR] from wearable wristband and participant number under *standing* activities.

| **Model coefficients^a,b^** | | | | | |
| --- | --- | --- | --- | --- | --- |
| Variable | B | Std. Error | β | t | p |
| (Constant) | -9517.293 | 3809.79 |  | -2.498 | 0.015 |
| Participant_number | -170.997 | 35.304 | -0.503 | -4.844 | 0.000 |
| Tskin | 305.227 | 100.885 | 0.323 | 3.025 | 0.004 |
| HR | 9.832 | 2.968 | 0.287 | 3.313 | 0.002 |

^a.^ Dependent variable: CO_2__Breathing_zone

^b.^ R²_adj_ = 0.594 (N = 65, p = 0.000)

**Table S7.** Adjusted R² value (relevant input variables) of MLR models with combined input parameters for IAQ exposure estimation during *combined* activities. The last row (colored as blue) indicates how much percent increase (%) was obtained in terms of estimation accuracy when using combined parameters compare to using a single IAQ parameter.

| **Combinations of parameters***  *(used as input variables)* | **Adjusted R² of composed MLR model (relevant input variables**)** | | | | |
| --- | --- | --- | --- | --- | --- |
|  | ***Combined activities*** | | | | |
|  | **CO_2_ estimation** | | **PM_2.5_ estimation** | | **PM_10_ estimation** |
| **Single IAQ** | 0.326  (Part_num, CO_2__Front edge of participant desk) | 0.861  (PM_2.5__Front edge of participant desk, Desk, Exhaust 1) | | 0.842  (PM_10__Front edge of participant desk, Desk, Exhaust 1) | |
| **IAQ + E4** | 0.474 (Part_num, CO_2__Front edge of participant desk, Tskin, HR) | 0.862 (PM_2.5__Front edge of participant desk, Desk, Exhaust 1) | | 0.845 (PM_10__Front edge of participant desk, Desk, Exhaust 1) | |
| **IAQ + PIRs** | 0.338 (Part_num, CO_2__Front edge of participant desk, PIR_Ceiling) | 0.863 (Part_num, PM_2.5__Front edge of participant desk, Desk, Exhaust 1) | | 0.843 (PM_10__Front edge of participant desk, Desk, Exhaust 1, PIR_ceiling) | |
| **IAQ + E4 + PIRs** | 0.49 (Part_num, CO_2__Front edge of participant desk, Tskin, HR, PIR_Wall, Desk) | 0.865 (Part_num, PM_2.5__Front edge of participant desk, Desk, Exhaust 1) | | 0.846 (PM_10__Front edge of participant desk, Desk, Exhaust 1) | |
| **Improvement of estimation accuracy**  *(Single IAQ vs combination of parameters, percent increase %)* | 50.3 | 0.5 | | 0.5 | |

^*^ IAQ: Stationary IAQ measurement, E4: Physiological measurement, and PIRs: Contextual measurement

** Part_num: participant number, Tskin: skin temperature, HR: heart rate

**Equation S1-S5.** Multiple regression equations for human exposure estimation to indoor air pollutants under both *sitting*/*standing* activities by using combinations of different parameters: IAQ monitor, wearable wristband, and PIR.

**1. *Sitting* activities**

**(i) Regression equation for CO_2_ exposure estimation**

$${CO}_{2,exposure}= -137.768{part}_{num}-1.653{CO}_{2,desk}+2.09{CO}_{2,exhaust}+ 872.547T_{skin}-293.286{PIR}_{wall}$$

$+227.358{PIR}_{desk}-29636.191$

(S1)

**(ii) Regression equation for PM_2.5_ exposure estimation**

$${PM}_{2.5,exposure}= 0.297{part}_{num}-1.302{PM}_{2.5,front edge of participant desk}+1.946{PM}_{2.5,desk}$$

$+ 1.764{PM}_{2.5,exhaust}+0.04HR-0.527{PIR}_{wall}-0.413{PIR}_{desk}-2.617$

(S2)

**(iii) Regression equation for PM_10_ exposure estimation**

$${PM}_{10,exposure}= 1.475{part}_{num}-1.465{PM}_{10,front edge of participant desk}+0.941{PM}_{10,desk}$$

$+ 1.876{PM}_{10,exhaust}-10.272T_{skin}+0.87HR+313.329$

(S3)

**2. *Standing* activities** *PM_10_ exposure estimation was excluded (low accuracy of a regression model)

**(i) Regression equation for CO_2_ exposure estimation**

$${CO}_{2,exposure}= -293.061{part}_{num}+0.713{CO}_{2,front edge of participant desk}+689.008{PIR}_{wall}+1466.035$$

(S4)

**(ii) Regression equation for PM_2.5_ exposure estimation**

${PM}_{2.5,exposure}=-4.156{PM}_{2.5,front edge of participant desk}+3.165{PM}_{2.5,exhaust}+1.036$

(S5)
